# Preferred Habitat of Breeding Birds May Be Compromised by Climate Change: Unexpected Effects of an Exceptionally Cold, Wet Spring

**DOI:** 10.1371/journal.pone.0075536

**Published:** 2013-09-18

**Authors:** Michael J. Whitehouse, Nancy M. Harrison, Julia Mackenzie, Shelley A. Hinsley

**Affiliations:** 1 Department of Life Sciences, Anglia Ruskin University, Cambridge, Cambridgeshire, United Kingdom; 2 Centre for Ecology and Hydrology, Natural Environment Research Council, Wallingford, Oxfordshire, United Kingdom; CNRS, Université de Bourgogne, France

## Abstract

Previous studies of the consequences for breeding birds of climate change have explored how their populations may respond to increasing temperatures. However, few have considered the likely outcome of predicted extreme conditions and the relative vulnerability of populations in different habitats. Here, we compare phenology and breeding success in great tits and blue tits over a 10 year period, including the extremely harsh conditions during spring 2012, at three sites in eastern England – mixed deciduous woodland, riparian and urban habitat. Production, measured as brood biomass, was significantly lower in 2012 compared with the previous 9 years, with the decrease in productivity relatively greatest in woodland habitat. Production was related to hatch delay, i.e. birds not initiating incubation immediately after clutch completion, which was more common in 2012 than in previous years. The best predictor of hatch delay was daytime temperature (not nighttime minimum temperature) and rainfall, which convincingly reflected low growth and activity of caterpillar prey. We found that birds breeding in riparian and urban habitats were less vulnerable to the extremes of weather than those breeding in mixed deciduous woodland.

## Introduction

Great tits *Parus* *major* and blue tits *Cyanistes* *caeruleus* are resident UK breeding birds occupying a great variety of habitats from oak woodlands to urban gardens [1]. Their propensity to use nest boxes and their relative robustness for small birds has made them ideal species to study and they are some of the most researched birds in the world [2,3]. Long term research on great tits in particular has been valuable in exploring the responses of breeding birds to climate change [4-11]. However, the majority of previous work has been conducted in woodland, with relatively little attention being paid to the potential consequences of changing climate on breeding birds in fragmented and urban habitats.

Although much of the UK was once densely forested, clearance began around 4000 BC and by about one thousand years ago, cover in England was reduced to about 15% [12]. Great tits and blue tits have adapted rapidly and now occupy a range of wooded habitats. More recently, their environment is undergoing further man-made modification due to climate change. Warmer springs have allowed great tits and blue tits to nest earlier in the year as warmer conditions have shifted the life-cycles of their dominant caterpillar prey [5,9,13,14]. However, in addition to temperature effects, it is also envisioned that climate change is likely to result in unpredictable rainfall patterns in the UK [15]. Rainfall and temperature are the main drivers of caterpillar reproduction and growth, and variability in their life cycles will inevitably impact birds that prey upon them [4,16].

The weather in the UK during the spring of 2012 was exceptionally cold and wet, the year being the wettest in England since records began in 1910 [17]. In this paper, we use 10 years of breeding data to examine the effects of this extreme weather on reproductive success in great tits and blue tits, and explore how their responses differed with habitat type.

## Methods

### Study sites

Nest boxes were monitored at three UK sites in Cambridgeshire in eastern England: Cambridge University Botanical Garden (CUBG; 52°12’ N, 0 ° 8’E), Cow Lane Nature Reserve (CL; 52°20’ N, 0° 9’W) and Brampton Wood Nature Reserve (BW; 52°19’ N, 0° 16’W). The three sites are in the same geographic area, experiencing similar temperatures and rainfall, but differ markedly in habitat structure and flora. The CUBG (40 ha) is a diverse urban habitat including native trees (~14%) and a comprehensive collection of plants from around the world [18]. CL (~85 ha) is a restored site of previous extensive gravel extraction alongside the river Great Ouse. Its riparian vegetation is dominated by willows ( *Salix* spp.) but includes several *Phragmites* reed beds. BW (131 ha) is mixed deciduous woodland dominated by common ash ( *Fraxinus* *excelsior* ), English oak ( *Quercus* *robur* ) and field maple ( *Acer* *campestre* ) along with some areas of conifers.

Nest boxes (CUBG: 20-44, CL: 50 and BW: 22) were monitored over a ten year period (2003-2012); in the CUBG the number of boxes changed during the time of the study with only 20 boxes from 2003 to 2005, increasing to 44 after 2005. Although nest box occupancy largely reflected population density, all three sites had some nest boxes (CUBG: 48% after 2005, CL: 24% and BW 14% respectively) with holes that excluded great tits.

### Bird data

Data were collected, for first nesting attempts only, at least weekly from the end of March to July each year. The following parameters were recorded for both species: nest completion, first egg date, clutch completion and size, hatching date, mean nestling body mass (excluding runts) and total brood biomass (including runts) at 11 days of age (day of hatching = 0), and the number of young fledged. Great tits and blue tits both usually lay one egg per day and begin incubation on the day the last egg is laid (which facilitates synchronous hatching). However, incubation can begin with the penultimate egg (or sometimes earlier) or it can be delayed beyond the date of the last egg. Hatch delay was calculated as observed minus expected hatch day, with the expected day being estimated as day of the last egg + 13, assuming that eggs usually hatch on the 14^th^ day of incubation [2].

Body mass was measured using a 50 g spring balance (*Pesola*) or a high precision pocket scale (*Satrue*), and nestlings were ringed with a uniquely numbered British Trust for Ornithology (BTO) ring. Runts (CL and BW) were defined as chicks too small to ring on day 11, and young not found dead in the box after the brood had left, were assumed to have fledged [19]. A different process for defining runts was required for the CUBG because of the size variation of nestlings at 11 days of age; frequency tables were generated using the masses of all nestlings from all boxes for both species and any nestling in the lowest 5% of these values were deemed to be runts. This calculation equated to any great tit nestling below 9.6 g in weight and any blue tit nestling below 4.4 g being excluded from the mean nestling body mass calculation [18].

### Weather data

Daily rainfall and temperature data were provided by CUBG. Although 25 km from CL and 29 km from BW, this was the closest data source with the detailed meteorological data required for this analysis. An index of spring warmth, the “warmth sum” was calculated as the sum of maximum daily temperatures from 1 March-25 April, as derived and used by previous investigators [5,20]. To consider hatch delay with respect to weather, mean temperature minimum and maximum along with rainfall was calculated for each individual nesting attempt for the week of clutch completion as well as the week prior to, and following clutch completion.

### Statistical analysis

Analysis of variance and covariance (ANOVA and ANCOVA), regressions and descriptive statistics, undertaken with Minitab Release 16, were used to compare breeding parameters between sites. General linear models (GLM) were used to investigate the influence of year, site, first egg date and hatch delay on three measures of productivity, i.e. brood biomass and mean nestling body mass at day 11, and the number of young fledged. First egg date and hatch delay were treated as continuous variables whereas year and study site were categorical. To investigate the effects of weather conditions, a best subsets regression was used that allowed for year and considered hatch delay in response to rain, daytime maximum and nighttime minimum temperatures during three critical weeks (the week of clutch completion, the week prior to this and the week following it). Best models were selected using the lowest Mallows’ Cp (a measure of goodness-of-prediction). Nests which survived to each stage of breeding were included in the analyses, hence sample sizes decrease through the season in most cases due to nest failures.

### Site access and study ethics

Access to the study sites was granted by the Director of the Cambridge University Botanical Gardens, and the land owners of Cow Lane Nature Reserve (Lafarge) and Brampton Wood Nature Reserve (the Wildlife Trust for Bedfordshire, Cambridgeshire and Northamptonshire). The study did not involve any endangered species. The fieldwork involved standard procedures as recommended by the BTO and everyone collecting data were licensed by the BTO and complied with the Codes of Practice as stipulated in the BTO’s Ringer’s Manual. No additional animal care approval was required.

## Results

### Relative impact of weather on productivity across habitats

**Figure 1:**
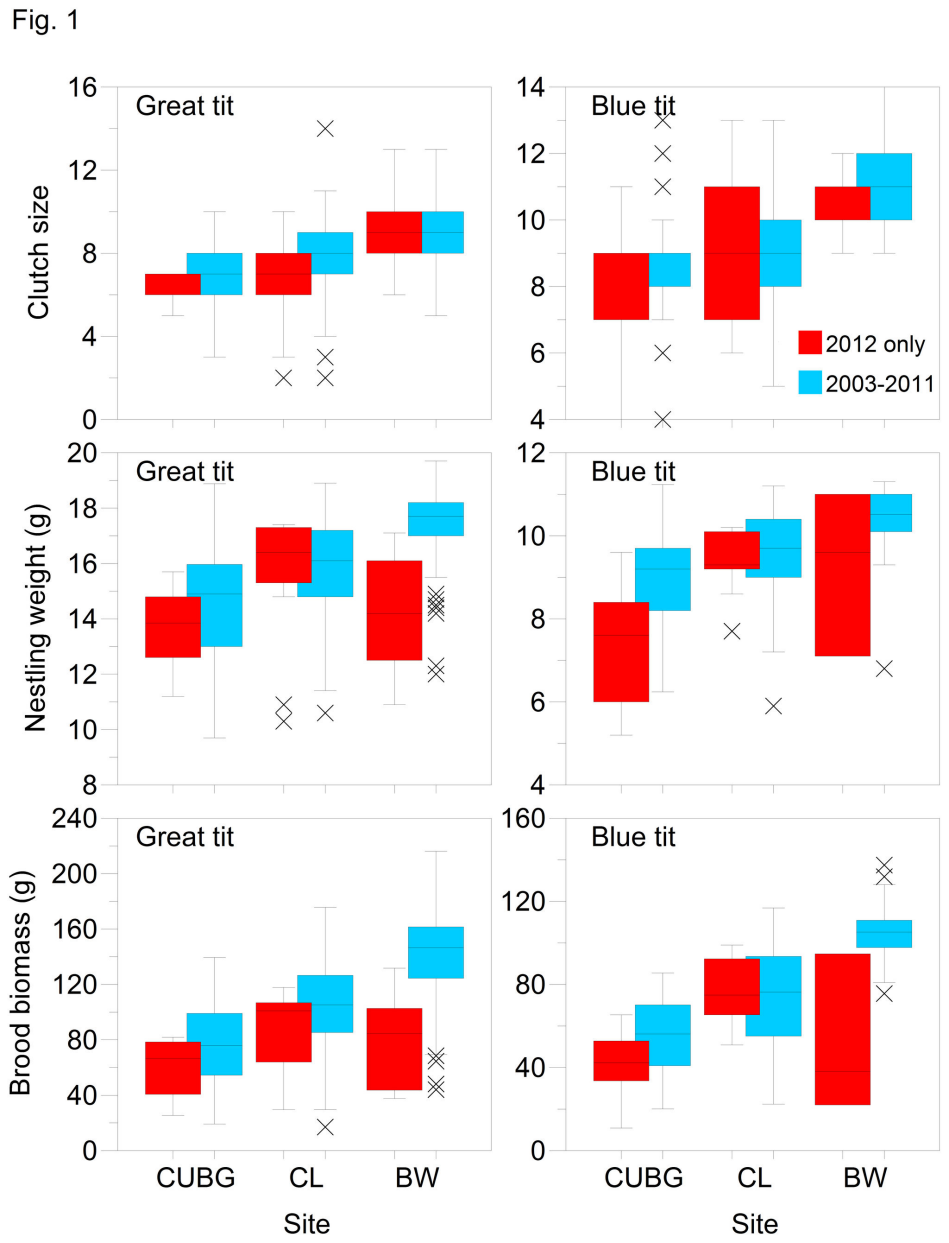
Median (bar), interquartile range (box), minimum, maximum and outlier values for clutch size, brood biomass and nestling weight for great tits and blue tits at Cambridge University Botanical Gardens (CUBG), Cow Lane Nature Reserve (CL) and Brampton Wood Nature Reserve (BW) during 2012 only (red) and 2003-2011 (blue).

The three study sites differed in clutch size and breeding success for both species (Table 1). While clutch size was fairly consistent within sites, mean nestling body mass and mean brood biomass illustrate the scale of the differences in productivity between the three sites and over the 10 years under consideration (Figure 1). For both species, year-to-year variability was least at BW. For great tits, prior to 2012 both measures were also consistently highest at BW and were usually lowest at CUBG (and especially so in 2005). However, in 2012 both measures of nestling mass were relatively low compared to the previous nine years and for BW, significantly lower (ANOVA, p <0.001 for both species). Although not quite so clear cut, blue tit mean mass and biomass were also relatively low in 2012 and especially so for CUBG. Although BW tended to produce heavier blue tit broods, biomass was significantly lower (ANOVA, p <0.005) at this site in 2012 than in previous years and mean nestling mass was the second lowest recorded during the 10 years.

**Table 1:** Overall breeding output of great tits *Parus* *major* (GT) and blue tits *Cyanistes* *caeruleus* (BT) in nest boxes at Cambridge University Botanical Garden (CUBG), Cow Lane Nature Reserve (CL) and Brampton Wood Nature Reserve (BW) during the years 2003 to 2012.

Species	Site	Total no. of clutches	Mean (SD) clutch size	Total no. of successful nests	Mean (SD) no. of young fledged
GT	CUBG	89	7.0 (1.3)	60	4.9 (1.8)
	CL	212	7.6 (1.7)	162	6.1 (2.1)
	BW	140	9.0 (1.5)	119	7.8 (2.1)
BT	CUBG	123	8.4 (1.6)	84	5.9 (2.1)
	CL	100	8.9 (1.6)	75	7.3 (2.3)
	BW	35	10.9 (1.3)	30	9.2 (2.6)

### Patterns in the timing of first eggs and occurrence of hatch delay

**Figure 2:**
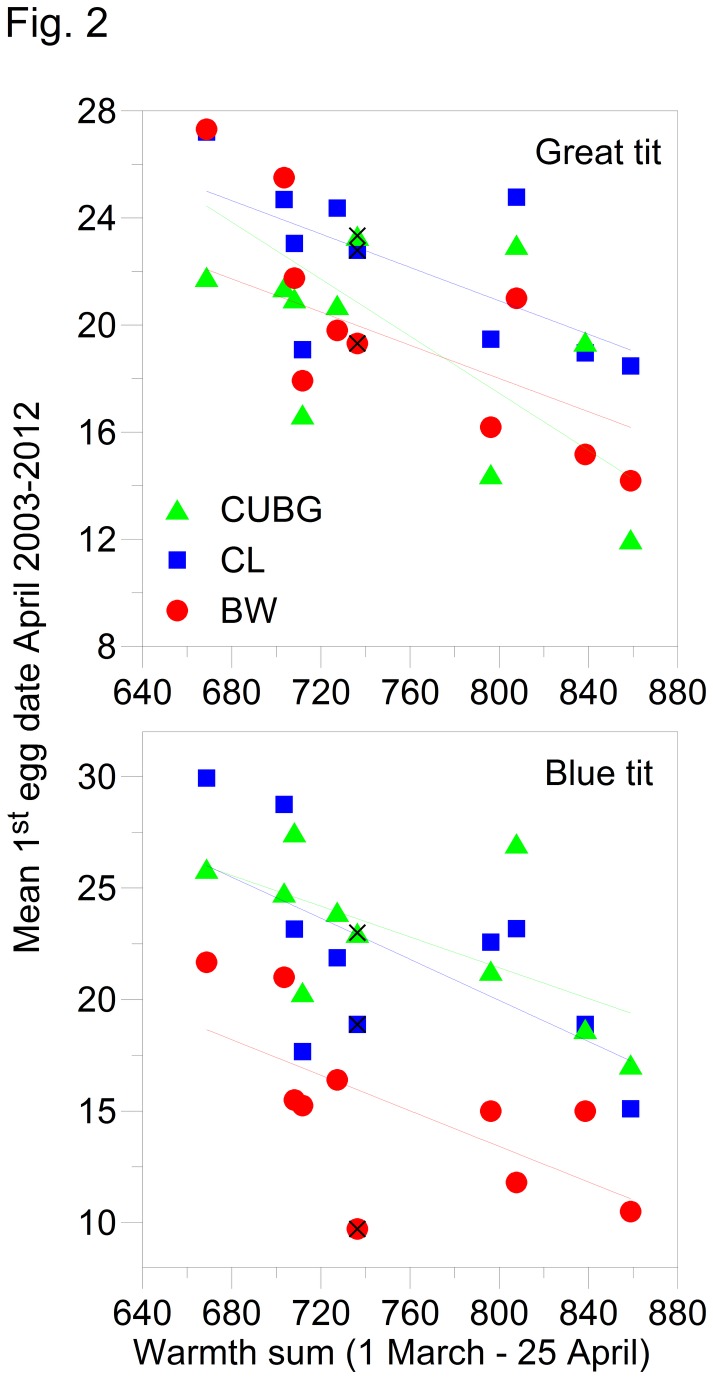
Mean first egg dates for great tits and blue tits at Cambridge University Botanical Gardens (green), Cow Lane Nature Reserve (blue) and Brampton Wood Nature Reserve (red) between 2003 and 2012. Symbols for 2012 are also marked (×). The warmth sum index is the sum of maximum daily temperatures from 1 March-25 April (see McCleery and Perrins 1998).

The general trend in the timing of breeding in relation to year-to-year variation in temperature followed the pattern documented previously [2,5,21], i.e. first eggs were laid earlier in warmer springs. However, there were significant differences between first egg dates at the three study sites (Figure 2). Over the 10 years of this study, great tits laid their first eggs significantly later at CL (ANCOVA p <0.05) compared with the other two sites. For blue tits, egg laying started significantly earlier at BW (ANCOVA p <0.05) compared with the other two sites. Overall, these trends are in keeping with the tendency of great tits to breed earlier in urban areas [18,22], whereas blue tits usually start before great tits in woodland [2,19]. In 2012 both species started to breed earliest at BW, and this was on average the earliest start for blue tits during the entire 10 year study. However, over the period 1 March -25 April for which the spring warmth sum was calculated [5,20] 2012 was by no means warm - indeed it falls in the lower half of the index range (Figure 2).

**Figure 3:**
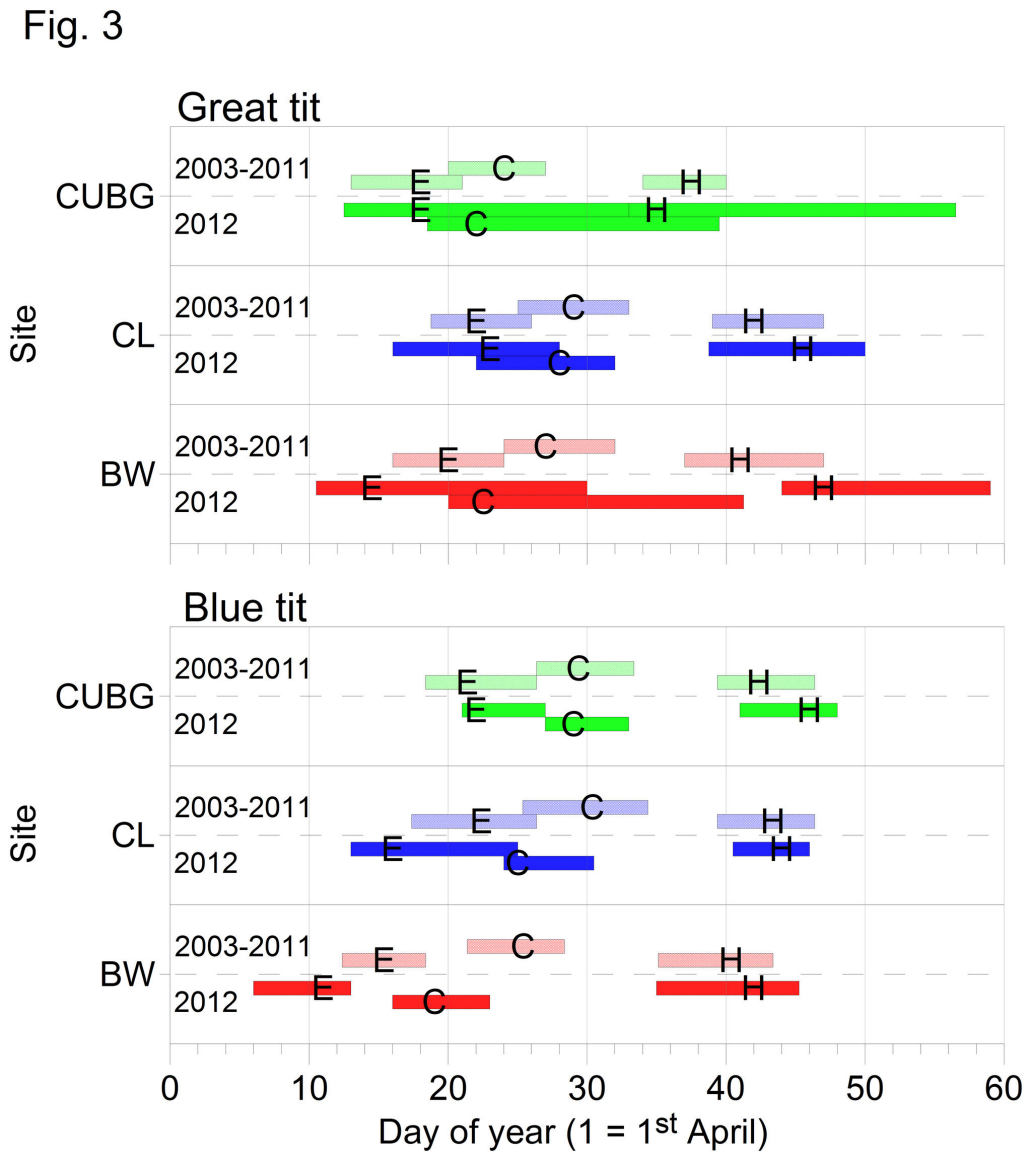
Average timing of nesting events for great tits (GT) and blue tits (BT) at Cambridge University Botanical Gardens (CUBG; green), Cow Lane Nature Reserve (CL; blue) and Brampton Wood Nature Reserve (BW; red). Symbols denote median dates for first egg (E), clutch completion (C; and earliest potential start of incubation) and hatching (H) and the bars show the interquartile range for the 9 year average 2003-2011 (hatched, upper), and 2012 only (solid, lower).

Despite the early first egg date for great tits at BW during 2012, they were the latest eggs to hatch (Figure 3). And although egg-laying by blue tits started earliest at BW in 2012 compared with CL and CUBG (~5 and 11 d respectively), average hatching was within ~4 days at all three sites. A comparison of the three sites (Figure 3) elucidates the components of this apparently extended breeding season at BW. On average, great tits laid their first eggs ~5 days earlier than in the previous 9 years for this site and thus the start of incubation and subsequent hatching could also have been early. However, the 2012 hatch dates at BW are 6 days later than the 9 year average and ~8 days later than they would have been if full incubation had begun as is typical on the day of clutch completion (Table 2). At CL, hatching was delayed a little, occurring ~3 days later than the 9 year average but within ~2 days of when they would have hatched with typical incubation (Table 2). At CUBG, hatching occurred at a date that suggested incubation had begun on the day of clutch completion which was 2 days earlier than the 9 year average and resulted in hatching that was ~12 days earlier than at BW. For great tits at BW in 2012, the period from first egg date to hatch day was ~32 days compared with 22 and 17 days at CL and CUBG respectively.

**Table 2:** Hatch delay of great tits *Parus* *major* (GT) and blue tits *Cyanistes* *caeruleus* (BT) at Cambridge University Botanical Garden (CUBG), Cow Lane Nature Reserve (CL) and Brampton Wood Nature Reserve (BW) during the years 2003 to 2011 and 2012.

Sp	period	CUBG		CL		BW	
		n	Median (range)	n	Median (range)	n	Median (range)
GT	2003-2011	68	0 (-2-3)	159	0 (-2-7)	109	0 (-2-5)
	2012	9	2 (-1-6)	17	2 (-2-9)	13	8 (0-13)
BT	2003-2011	98	0 (-2-3)	77	0 (-1-9)	26	1 (-1-5)
	2012	19	2 (0-6)	7	2 (0-14)	6	8.5 (7-10)

Hatch delay was calculated as observed minus expected hatch day, with the expected day being estimated as day on which last egg was laid + 13.

Similarly for blue tits, egg-laying during 2012 was 4 days earlier than the 9 year average at BW and clutch completion was proportionally early compared with previous years (Figure 3). However, despite the differential between first laying dates, on average, hatching occurred within 4 days at all 3 sites. As with great tits, although delays with incubation and hatching were minimal in the previous 9 years (Table 2), during 2012 there was an average hatch delay of 2 days for blue tits at CL and CUBG but >8 days at BW. Also noteworthy was that all blue tits at BW delayed their hatching by at least 7 days which did not occur at the other sites or for great tits and had not occurred in any of the previous 9 years.

At BW during 2012, egg laying was interrupted by 1-2 days in the case of 2 out of 7 blue tits and 1 out of 17 great tits but the majority of birds laid one egg per day until their clutch was completed and then left cold eggs in the nest to be incubated at a later date. This prolonged stalling of the nesting cycle was unprecedented during the 10 year period of this study at any of the three sites. Typically, full-time incubation usually started for both species within a day or two of clutch completion and hatching usually occurred ~14 d after the start of incubation.

### Influence of laying dates and hatch delay on productivity across habitats

In the GLMs investigating the influence of first egg date and hatch delay on productivity, year and site were always significant predictors (Table 3). The best fit models were for brood biomass with R^2^ estimates of 52% and 47% for great tits and blue tits respectively. First egg date was significantly, negatively related to brood biomass for great tits and blue tits while hatch delay was significantly, negatively related to brood biomass for great tits. First egg date and hatch delay were significantly, negatively related to the number of young fledged for both species. Both variables were poor predictors of mean nestling weight in both species. Overall, the later the first egg date, and the longer the hatch delay, the lower the brood biomass and the number of young fledged.

**Table 3:** General linear models relating three measurements of productivity (brood biomass and nestling mean weight on day 11, and number of fledglings) to year, site, 1^st^ egg date and hatch delay during 2003-2012.

Sp	Response	R^2^	Year		Site		1^st^ egg date		Hatch delay	
		%	F	*p*	F	*p*	F	*p*	F	*p*
GT	Brood biomass	52	2.3	**0.016**	117	**<0.001**	29	**<0.001**	53	**<0.001**
	Nestling mean weight	32	3.6	**<0.001**	50	**<0.001**	0	0.970	2.0	0.161
	Number of fledglings	35	2.3	**0.016**	61	**<0.001**	17	**<0.001**	12	**<0.001**
BT	Brood biomass	47	2.9	**0.003**	34	**<0.001**	17	**<0.001**	3.7	0.056
	Nestling mean weight	32	3.8	**<0.001**	24	**<0.001**	5.2	0.024	3.7	0.055
	Number of fledglings	34	2.9	**0.003**	19	**<0.001**	12	**<0.001**	7.8	**0.006**

Significant results (*p* <0.05) for 1^st^ egg day and hatch delay (whose coefficients were negative) are emboldened.

However, this was not the case for great tits at BW during 2012 when the pattern reversed, all measures of productivity were positively, but not significantly, related to first egg date (linear regression: biomass R^2^ = 27%, F = 3.7, p = 0.082; number of young fledged R^2^ = 21%, F = 3.7, p = 0.075; mean nestling weight R^2^ = 28%, F = 3.8, p = 0.08). For great tits at CL during 2012 all measures of productivity were similarly positively related to first egg date and not significant (linear regression p ≥0.164). However, great tit productivity at CUBG followed the overall trends and was negatively related to first egg date (linear regression: biomass R^2^ = 68%, F = 13, p = 0.012), but not significantly so for mean nestling weight and number of young fledged (p ≥0.306). For blue tits during 2012, productivity at CL and CUBG was generally negatively related to first egg date as in the overall analyses above, albeit non-significantly for this year (linear regression p ≥0.098). However, as with the great tits, this relationship was positive at BW, although not significant (linear regression p ≥0.364).

We considered the relationship between first egg date and hatch delay. For great tits during 2003-2011 hatch delay was rare, and there were no consistent trends with the longest hatch delay peaking 20-30^th^ April at all three sites. However, for great tits during 2012 delayed incubation and thus hatch delay was frequent; although there was no consistent trend at CUBG, first egg date and hatch delay were significantly negatively related at CL (linear regression R^2^ = 26%, F = 4.8, p = 0.045) and strongly, negatively related at BW (linear regression R^2^ = 70%, F = 26, p <0.001). For blue tit the relationship between first egg date and hatch delay was negative at the three sites but was not significant (linear regression p ≥0.125), and again the relationship between first egg date and hatch delay during 2012 was generally negative but was not significant (linear regression p ≥0.07)

### Vulnerability to extreme weather across habitats

**Figure 4:**
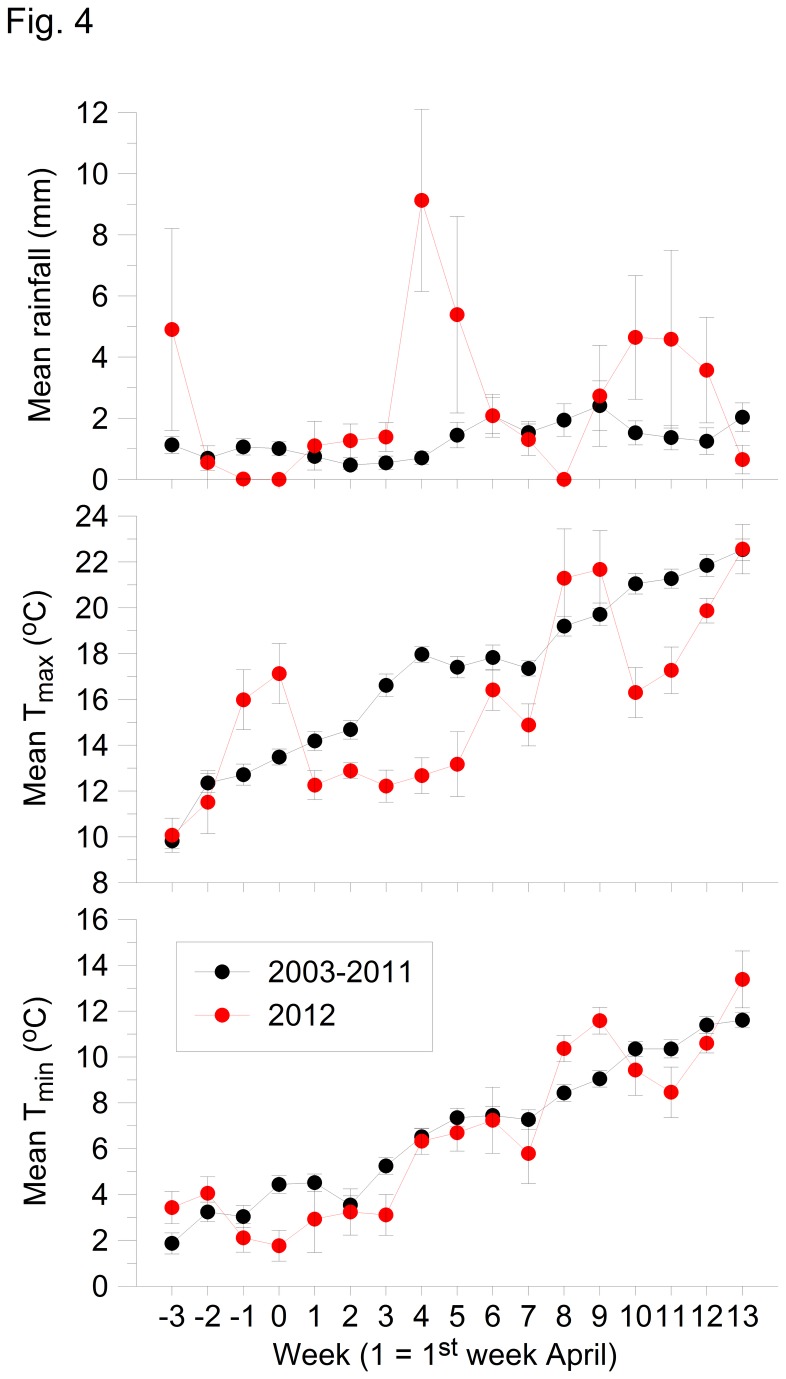
Mean (and standard error) weekly rainfall, temperature maximum (T_max_) and minimum (T_min_) during March-June (week 1 = 1^st^ week of April) for 2003-2011 (black) and 2012 only (red).

The weather in the UK during the spring of 2012 was exceptionally cold and wet [17]. Potentially worse for the birds at our Cambridgeshire study sites was a warm and dry period during the last two weeks of March which may have encouraged early breeding (Figure 4). However, while the first three weeks of April were marginally wetter than average, the following two weeks of 2012 were significantly wetter (ANOVA p<0.001) than in the previous 9 years contributing to a record-breaking wet spring and summer in the UK [17]. Furthermore, daytime temperature maximums (T_max_) from the first week of April through to the second half of May were significantly lower (ANOVA p<0.001) than in the previous 9 years. Similarly, nighttime temperature minimums (T_min_) were below average from the last week of March through to the third week in April.

Hatch delay was significantly related to weather conditions, especially temperature (Table 4). The correlation was particularly strong for BW where hatch delay was longest in both great tits and blue tits (R^2^ = 72% and 84% respectively). For all models, hatch delay was negatively related to temperature and positively related to rainfall. The best model for each species at each site was obtained by using 3 of the 9 possible predictor variables (rain, day and night temperatures during each of the 3 critical weeks). Of the total of 18 factors identified for both species at the 3 sites, the most frequently selected was T_max_ (n = 8) which also represents daytime conditions. T_min_ (nighttime) was the next most frequent (n = 6) followed by rainfall (n = 4).

**Table 4:** Best subset models to predict hatch delay for great tits *Parus* *major* (GT) and blue tits *Cyanistes* *caeruleus* (BT) at Cambridge University Botanical Garden (CUBG), Cow Lane Nature Reserve (CL) and Brampton Wood Nature Reserve (BW) from mean weekly maximum daily temperature (T_max_; ° C), minimum daily temperature (T_min_; ° C), and rainfall (rain; mm).

Sp	Site	R^2^	M. Cp	S	E			C			I		
					T_max_	T_min_	rain	T_max_	T_min_	rain	T_max_	T_min_	rain
GT	CUBG	15	0.5	1.24		X		X				X	
	CL	32	1.9	1.33		X				X		X	
	BW	72	4.4	1.54	X			X					X
BT	CUBG	48	1.6	1.04	X				X	X			
	CL	25	7.5	1.96	X						X	X	
	BW	84	1.5	1.44	X			X		X			

The pertinent weeks were: i) the week before clutch completion, i.e. during egg laying (E); ii) the week of clutch completion when full incubation should start with the laying of the last egg (C); iii) the week after clutch completion which typically would be the second week of incubation (I). The best models were obtained with 3 of the 9 possible predictor variables; models with the lowest Mallows Cp (M. Cp) values and standard error of regression (S) were selected. For all models, hatch delay was negatively related to temperature and positively related to rainfall.

**Figure 5:**
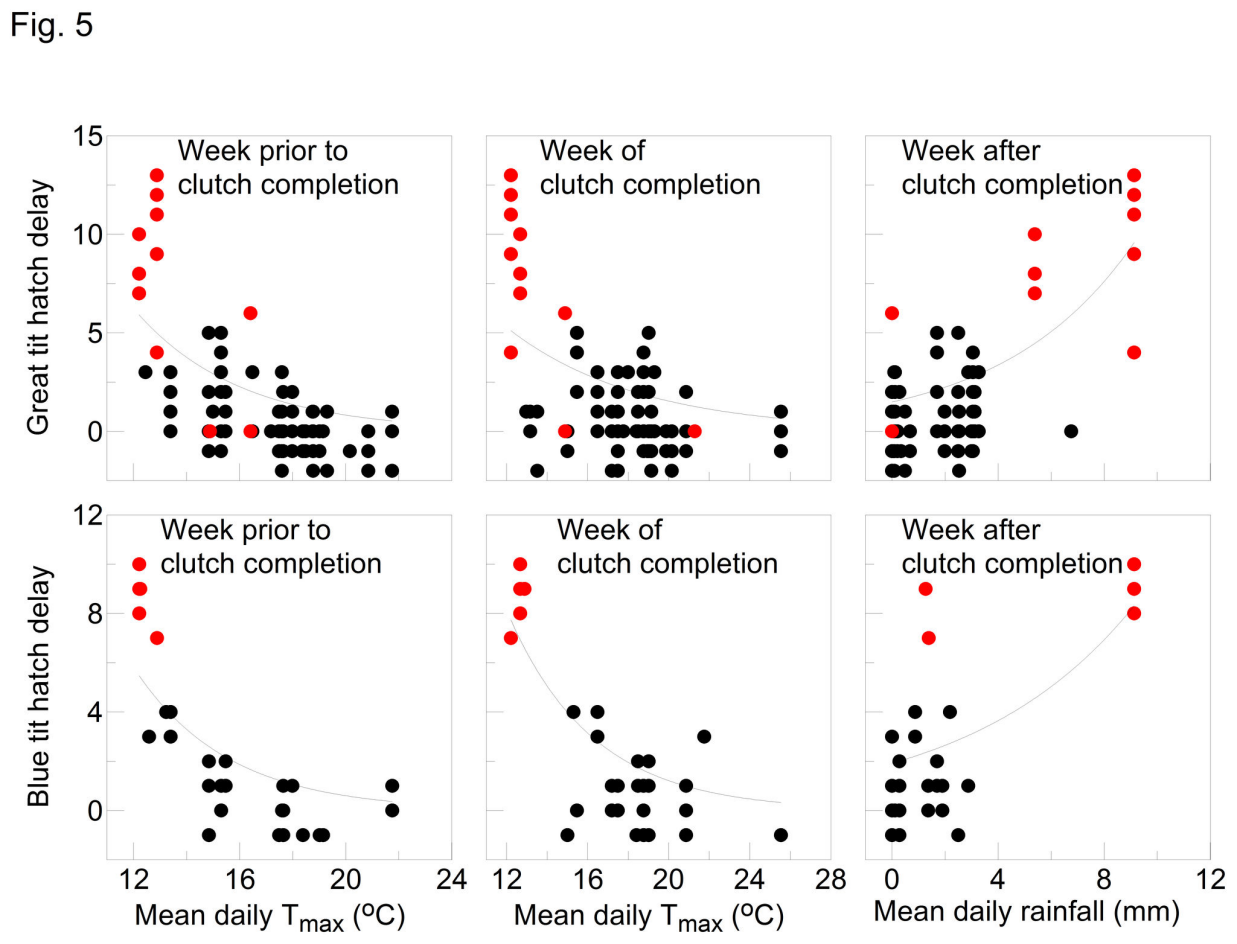
Hatch delay for great tits and blue tits at Brampton Wood Nature Reserve in relation to mean daily temperature maximum (T_max_) and rainfall (the best predictor weather variables) during the week prior to clutch completion, the week of clutch completion, and the week after clutch completion. Symbols represent individual nests during 2003-2011 (black) and 2012 only (red).

**Figure 6:**
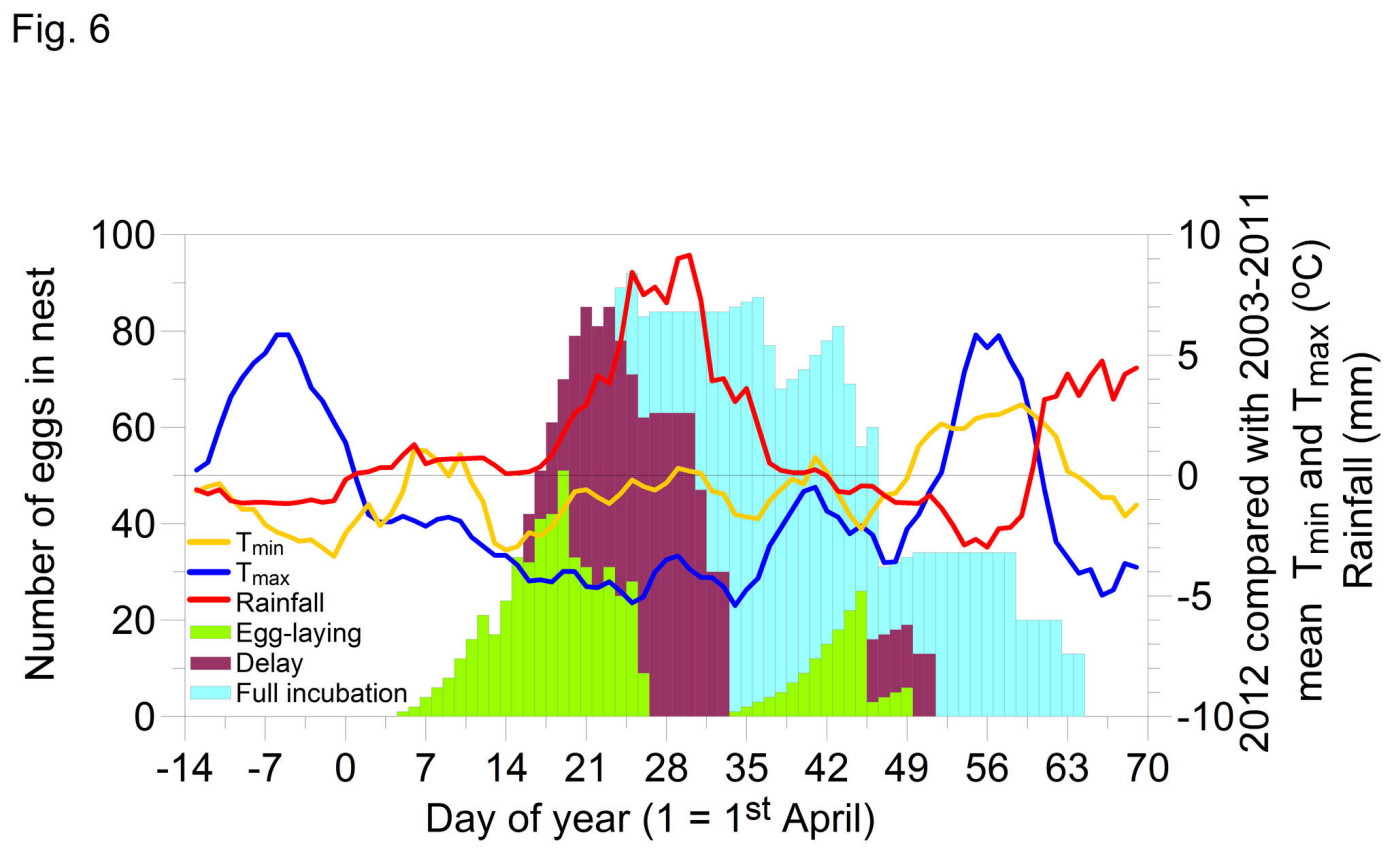
The progression of egg-laying, delayed or partial incubation, and full incubation for great tits at Brampton Wood Nature Reserve during 2012. Overlaid is the difference in night-time and daytime temperatures and rainfall between 2012 and the average for the preceding nine years.

For BW, there was more of a continuous relationship between hatch delay and temperature with 2012 representing extreme conditions (Figure 5). The most acute challenge arguably was the result of rainfall; 2012 represented an extreme compared to preceding years due to the exceptional volumes of rain involved. The hatch delay was particularly prolonged for great tits at BW, and this is brought into focus if we consider the different stages of egg presence and development in the nest (laying, delay prior to incubation and incubation) against weather conditions prior to and during these stages (Figure 6). During the last two weeks of March, although nighttime temperatures were below average, daytime temperatures were well above average and rainfall was below average. Egg-laying commenced during the first week of April and peaked during the third week by which time nighttime temperatures were below average, daytime temperatures had plummeted and rainfall was building towards a record high. There was a substantial delay before the females began full-time incubation which peaked at the beginning of May (day 31) by which time night temperatures were nearing average and rainfall was diminishing. However, daytime temperatures remained below average until the fourth week of May (day 52). Note also that as the last 20 or so eggs were hatching, temperatures dropped below average once more and rainfall increased considerably.

## Discussion

This study shows that 2012 was a poor year for breeding great tits and blue tits, but further indicates how weather interacts with habitat to determine breeding success. The weather in 2012 was extreme [17], and for both species total productivity (brood biomass) was significantly lower in 2012, most strikingly at BW, the site most representative of preferred woodland habitat. Brood biomass is a good indication of the total energy available for reproduction and indicates that the cold, wet conditions in 2012 limited the resources available for brood rearing. The mean weights of nestlings were significantly lower in 2012; nestling weight at fledging is positively related to likely recruitment [23,24] and the low nestling weights in 2012 at BW and CUBG suggest lower likely recruitment. However, where there were small brood sizes (so that more energy was invested in each individual offspring) some of the nestlings reached healthy weights. Particularly for great tits, clutch sizes were small at CL and CUBG in 2012, and nestling weights were similar (CUBG) or greater (CL) than at BW.

Compared to blue tits, great tits appeared to be more vulnerable to the food shortages in BW, and this may relate to their larger mass (18 g for great tit compared with 11g for blue tit) [25]. Blue tits, being approximately 60% the mass of great tits, differ in their foraging requirements and appear to be less vulnerable during times of food shortages. Our observations of breeding performance are strongly suggestive of a fundamental difference between BW and the other study sites, with BW birds dependent upon the spring peak in caterpillars, while CL and CUBG birds use a broader resource base. In the urban habitat of CUBG, there is a large diversity of non-native plants and a heterogeneous configuration of flora, which is likely to result in low caterpillar density [26] and abundance [27]. Overall, blue tits have been found to be more selective foragers than great tits during the breeding season in CUBG, feeding more frequently in native deciduous trees and shrubs where they are likely to find more invertebrate food for their nestlings [18]. Visser et al. [28] argued that quantifying food resources is essential to establishing an understanding of phenology. However, the extremely diverse resources used by populations in riparian and urban habitats are not readily quantified (due to the large numbers of plant species), and breeding pairs may vary in their dependence on Lepidopteran or other insect prey across small spatial scales. Our results showing poor performance in woodland in 2012 supports our impression that there is lower dependence on one or few prey taxa in marginal and urban habitats.

The breeding season is controlled by a hierarchy of factors, first photoperiod and then temperature [29], with temperature a good predictor of bud burst and caterpillar abundance [16,28]. The warm conditions in March 2012 set the breeding season in motion, but then birds found themselves laying eggs in temperatures near freezing. Daytime April temperatures were up to 5 degrees below average in 2012. Breeding birds would be affected both because of cold conditions increasing energy requirements and less food being available [24].

In 2012 the response to cold conditions was delayed incubation, which created a lag in hatching and fledging. The reason may have been the condition of the laying females, with incubation having energetic consequences [5,30]. However, the outcome was that critical periods for feeding young and fledging chicks were delayed until more prey was, potentially, available. The caterpillar season is prolonged in colder springs [31], with growth rates and developmental times correlated with ambient temperature [32]; for example, for *Operophtera* *brumata* a common prey of birds feeding in woodland habitat, the Q_10_ = 3 [4]. Thus given the likely effects of the cold conditions on caterpillar availability, the birds at BW probably faced an unprecedented food shortage. Moth recording in our study region showed that 2012 was a poor year with fewer moth species and numbers of individuals recorded than normal, caterpillars being affected by the cold but also perishing in the heavy and frequent rain (Barry Dickerson, Huntingdonshire Moth Recorder, pers. comm.). Our exploration of hatch delay was informative, elucidating how weather disrupted egg-laying and incubation. Our results show that the most significant predictor of hatch delay was not low nighttime temperatures (T_min_) but instead low daytime temperatures (the daily T_max_). Furthermore, although temperature was more significant than rainfall in this study, heavy rainfall can wash caterpillars off leaves thus increasing their scarcity for leaf-foraging predators [33].

Clutch sizes were larger in the more favourable habitat of BW, and the physiological stress of producing the large clutches may have explained some of our observations of mortality within broods. Food stress in females during egg-laying can result in eggs of diminishing mass which in turn results in a gradation of nestling sizes on hatching [2]. Competition for scarcer caterpillars between nestlings of different sizes would favour the larger birds and likely result in the death of the smaller ones. The mean weights of nestlings were significantly lower in 2012 and highly variable, with broods at BW showing high variation in the size of nestlings of the same age, even in the same nest. The timing and size of clutches are shaped by natural selection [34] with a pattern identified in which birds laying early are at a selective advantage [23,35]. If constraints during egg-laying become more severe, the birds have the option of trading-off the costs of producing eggs early against initiating incubation before clutch completion [10,36], with asynchronous hatching of the chicks a possible consequence [6].

Once egg-laying is initiated, there is said to be little flexibility in the progress of breeding from incubation, through to nestling provisioning and fledging [37]. Selection for the timing of egg-laying is thus considered of paramount importance, the sole factor determining the timing of nestlings relative to the peak in resource availability. A response has been shown in great tits as the result of changing phenology associated with climate change, with increased strength of directional selection on the timing of breeding [38]. However, if delayed incubation is a viable option, the birds may have more flexibility in the event of catastrophic change in conditions such as those observed in 2012.

There is growing evidence for adaptive plasticity in blue tits [39] and great tits [4,5,9] permitting the alignment of hatching and the demands of feeding their brood with the peak availability of caterpillar prey. The birds are more able to delay hatching than to accelerate it through changes in clutch size and incubation [4,5] and this has pushed the date of first eggs earlier. Strong selection may result in microevolution in laying dates, or in phenotypic plasticity, and whether this succeeds in aligning reproductive effort with resource availability depends upon how rapid climate changes may be, and whether the different elements (e.g. bud burst and caterpillar growth) are responding at similar rates [10,11,16].

There exists a challenge in identifying the extent to which observed incubation delays represent females constrained by food shortages or adaptive plasticity, and our data comparing blue tits and great tits suggests that constraints on females may be part of the explanation of observed laying and incubation behaviour. Arnold et al. [40] showed that a non-heritable trait (nutritional state) affected fitness. Clutch size may also be under directional selection, but it is similarly affected by nutritional state; such proximate constraints may be correlated to fitness. Decisions of individuals – in the present study, delays in hatching as the result of delay in initiation of incubation – show how a proximate mechanism may contribute to the dynamics of selection, with implications for evolutionary episodes [41] and a potentially rapid response to climate change.

Where populations are under stronger selection, for example dependent on a more specialised diet, then the speed of evolution will be faster, responding to the rapid adaptive response of insect phenology to climate change [16,42]. If we consider brood biomass and mean nestling weights, we see that 2012 was worse for BW but relatively less poor for the other sites (Figure 1). We believe this outcome for the woodland breeders may be explained by the constraints of specialising on a relatively narrow prey base, chiefly Lepidopteran larvae. In contrast, a greater variety of habitats at CL (for example, the most successful blue tits foraged in *Phragmites* reeds, pers. obs.), and CUBG (urban mosaic habitat) allowed these more generalist foragers to switch between prey, leaving them less vulnerable.

Changing temperatures have been identified as affecting the timing of the initiation of breeding in great tits, an empirical example of a biological/ecological signature of climate change [6,8,28,42]. The trends in spring temperature may be a potent selective force on breeding birds [6,39,43], but our analysis is illustrative of the complex consequences for ecological processes as the result of climate change [44,45]. Not only are milder winters and springs predicted, but also unpredictable storms [15,46]. Charmantier et al. [9] noted that while great tits were showing adaptive plasticity in response to warmer springs, they would not be very efficient in developing some alternative plasticity to different environmental conditions or a different rate of change. Plasticity is related to environmental predictability, an important adaptation for small scale environmental heterogeneity [39], but the birds are not able to respond to wildly unpredictable events such as those of spring 2012. Our observations suggested that there were responses, notably delays in the initiation of incubation, which could have fitness consequences if the peak in prey availability was shifted to later in the spring. However, conditions were not good for most of the spring of 2012. The weather was again rainy and cold in June at the time of fledging. It would appear that as a result of unprecedented cold, rainy conditions, 2012 was a very bad year for tits for two critical stages important for success - provisioning in the nest and fledging.

Breeding success of both great tits and blue tits has been shown to be higher in deciduous woodland compared to other habitats with breeding performance particularly problematic in urban habitat [18,47]. Studies of energetics in the sites presently being considered have shown that breeding birds work much harder to produce fewer offspring in urban habitats as compared to woodland [48,49]. The present study found all of the birds struggling to raise young in 2012, but the variable performance of birds in riparian and urban habitats was not very different from a typical year, whereas the early laying birds breeding at BW performed particularly poorly. We predict that recruitment across these habitats will be low, but with a larger relative production of recruits in marginal habitats, and among the birds laying later in the season.
